# Screening for Anal Dysplasia in Adolescent and Young Adult Men Who Have Sex With Men Living With HIV, a Review of Current Recommendations

**DOI:** 10.3389/fped.2022.875184

**Published:** 2022-04-06

**Authors:** Jessica Addison, Carly Guss, Susan Fitzgerald, Elizabeth Woods

**Affiliations:** Division of Adolescent/Young Adult Medicine, Boston Children's Hospital, Harvard Medical School, Boston, MA, United States

**Keywords:** adolescent, HIV, Sexually Transimitted Diseases (STDs), anal dysplasia screening, anoscopy

## Introduction

The incidence of anal dysplasia caused by the human papilloma virus (HPV) is increasing in men and can be a precursor to the development of anal cancer (1). Oncogenic HPV types are believed to be the causative agent in up to 90% of anal cancers. In men who have sex with men (MSM) living with the Human Immunodeficiency Virus (HIV), the incidence of anal cancer is even higher (45.9 per 100,000) vs. (5.1 per 100,000) in MSM not living with HIV (2). Additionally studies have shown that the prevalence of having any type of anal HPV type can be as high as 92.6% in MSM living with HIV (3). There are well known screening guidelines for cervical cancer in women starting at age 21. However, screening for anal dysplasia caused by HPV in high risk adolescent/young adult (AYA) population (<26 years old) such as MSM living with HIV is not standardized or universally recommended even though the risk of anal cancer in this population can be as high as two times the rate of cervical cancer in women (4). Anal cytology has been recommended by several research groups for screening at-risk populations for anal cancer based on the guidelines of cervical screening; however, there are no universal screening guidelines for MSM living with HIV <35 years of age (5, 6). The purpose of this opinion paper is to review current recommendations for screening for anal dysplasia in AYA MSM living with HIV, with implications for clinical practice and research.

### Variation in Current Guidelines and Recommendations

**Figure 1:**
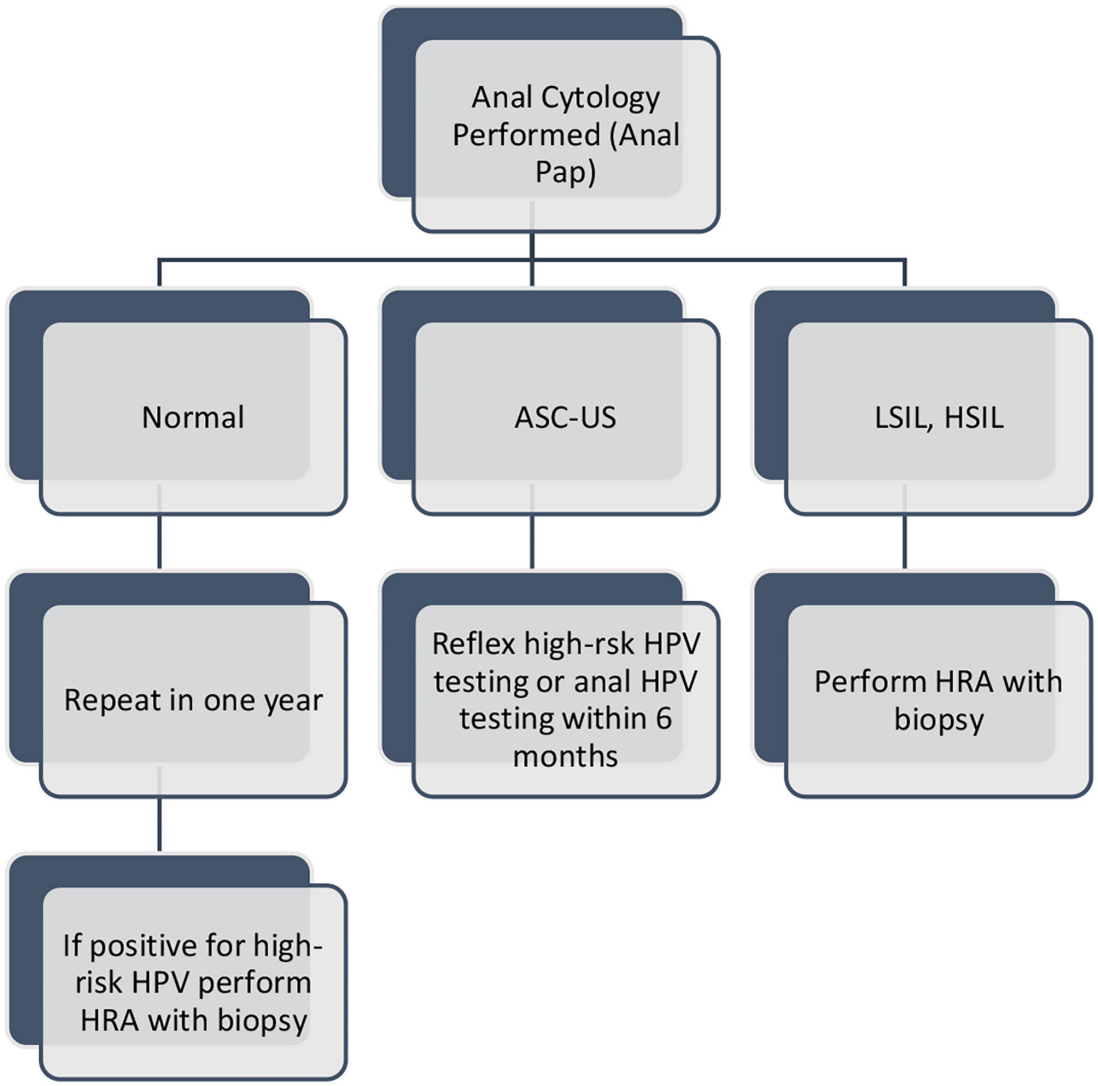
Anal cytology Follow-up Guidelines*. Key: ASC-US: atypical squamous cells of undetermined significance; LSIL: low-grade squamous intraepithelial lesions; HSIL: high-grade squamous intraepithelial lesion, HRA: high resolution anoscopy, *For MSM ≥ 35 years old. Adapted from: New York State Department of Health AIDS Institute: Clinical Guidelines Program: Screening for Anal Dysplasia and Cancer in Adults with HIV. https://www.hivguidelines.org/hiv-care/anal-cancer.

The 2020 Centers for Disease Control and Prevention (CDC) STI treatment guidelines does not recommended routine anal Papanicolaou test (Pap test) screening for any specific sub-group due to insufficient data (7). The New York State Department of Public Health AIDS Institute has published guidelines recommending annual digital anal rectal exam for all patients living with HIV who are ≥35 year old (8). For those ≤35 anal symptoms that would suggest anal dysplasia should be evaluated (8). The 2020 HIV Medicine Association of the Infectious Diseases Society of America guidelines state the “periodic anal cytology by anal Pap test should be performed if access to referral and high resolution anoscopy is available” (9). Table 1 summarizes the variation in current screening recommendations in more detail and highlights that there is only one applicable to AYA MSM living with HIV. Figure 1 outlines follow up recommendations if anal cytology is performed.

**Table 1:** Recommendations in screening for anal dysplasia in MSM living with HIV.

**Society/Institution**	**Age specification**	**Recommendation**
New York State Department of Health AIDS Institute (8)	>35 years old	Annual digital anal rectal exam Discuss smoking cessation to decrease risk of anal cancer Annual anal pap testing (If abnormal refer for high resolution anoscopy)
New York State Department of Health AIDS Institute (8)	<35	Evaluate for any signs or symptoms that suggest anal dysplasia (ex: anal itching, bleeding, pain)
European AIDS Clinical Society (10)	No	Digital anal rectal exam +/- anal cytology every 1–3 years Referral to anoscopy if anal cytology is abnormal
HIV Medicine Association of the Infectious Diseases Society of America guidelines (9)	No	Persons with a history of receptive anal intercourse should have an anal Pap test if there is access to appropriate referral for follow-up
Centers For Disease Control and Prevention (11)	No	A digital anorectal examination should be performed in individuals living with HIV and MSM not living with HIV but who have a history of receptive anal intercourse Cytology-based screening program should only be performed if referrals to high-resolution anoscopy and biopsy are available

There is currently a paucity of data in the AYA MSM population regarding the natural history of HPV infection, the development of anal dysplasia, and the efficacy of anal Pap tests. The majority of studies analyzing the incidence and prevalence of anal dysplasia are in MSM living with HIV ≥35 years old to reflect New York current screening guideline. Studies that include <26 participants who were found to have abnormal Pap tests had a median age of 40–50 years highlighting the lack of representation of younger demographics. A recent meta-analysis that stratified the prevalence of anal HPV by age and included a younger demographic (AYA MSM living with HIV 15–26 years old) highlighted the need for continued research in this field; specifically studies that include individual level data so risk and patterns of anal dysplasia by age can be analyzed (12). Barriers to acquiring this data in this population include: lack of universal screening guidelines for anal dysplasia, challenges with collecting information on sexual practices in this demographic due to provider discomfort and perceived stigma that patients may have about disclosing sexual activity to providers. All of these factors highlight the need for additional research to provide recommendations for the use of anal Pap tests for screening in younger individuals.

### Risk Factors and Trends for Anal Dysplasia in AYA MSM Living With HIV

HIV is an independent risk factor for anal dysplasia. Additional risk factors for anal dysplasia in MSM living with HIV include engaging in receptive anal intercourse, and low CD4 counts (<200 cells/μL) (12). AYA MSM living with HIV are having their sexual debut at younger ages, have multiple sexual partners, and studies have shown antiviral adherence rates are lower ranging from only 28.3 to 69.8% (13). All of these factors can increase the risk of anal dysplasia

A meta-analysis performed by Wei et al. (12) showed that AYA MSM living with HIV aged 15–18 years old had an anal HPV16 and other high risk HPV strains prevalence of 5.6% and 58.3% respectively. The prevalence increased significantly to 28.8% and 74.4% for HPV 16 and high risk HPV in those who were 23–24 years of age (12). Factors that may be contributing to this increase include impaired clearance of anal HPV in this age group or behavioral factors such as an increase in sexual partners with age (14). Despite the fact that prevalence of anal HPV increases with age within this demographic, there are still no universal screening recommendations.

## Discussion

While awaiting potential universal screening guidelines in this younger population, the discussion of anal dysplasia and anal cancer should be part of routine care in AYA MSM living with HIV. Reasons for delayed or missed opportunities for counselling include lack of guidelines, slow progression of anal dysplasia to anal cancer, low incidence of anal cancer in AYA, and provider unfamiliarity with risk factors for anal dysplasia. In women, progression to cervical cancer can take anywhere from 3 to 7 years for high-grade cervical changes. Despite the slower progression in men, counselling about anal dysplasia and HPV should be discussed at routine medical visits. Studies have highlighted the lack of provider awareness as a barrier to counselling and educating patients around risk factors for anal dysplasia and that most AYA MSM living with HIV did not know about HPV infection and its connection to anal cancer (15). Cervical cancer screening in women and the risks of HPV infection is routinely discussed during primary care visits regardless of a women's sexual history well before age 21 to prepare and educate them on routine screening. Similarly, the HPV vaccine is more consistently addressed for young women who also have higher immunization rates compared to young men, 53.6% vs. 27% respectively (16). Therefore, primary care physicians who provide care to AYA MSM living with HIV should consider the potential benefits of screening for anal dysplasia and find ways to incorporate it into their routine clinical practice.

Although anal cancer is rare before the age of 26 in AYA MSM living with HIV, more research is needed in the adolescent population to accurately depict the incidence and prevalence of anal dysplasia. There have been recommendations to make screening for anal dysplasia with anal Pap test a shared decision making process between the providers and patients and should be performed if requested (17). Providers should inquire about number of sexual partners, type of sexual intercourse (receptive vs. insertive anal sex), HPV vaccinations status, and age of sexarche at visits where risk of anal dysplasia is being assessed. Due to the lack of studies investigating the incidence of anal dysplasia in this specific population and the potential for complications as a result of anal dysplasia and HPV pathogenesis, screening in this younger age range (<35) should be considered based on risk factors to slow disease progression and prevent malignancy. Additionally studies have shown that anal dysplasia and cervical dysplasia caused by HPV peaks at similar ages in AYA MSM living with HIV and women (around age 25) (12). HPV vaccine recommendations are now the same across genders starting as young at age 9 through 26 years of age and should be discussed equally. It is also imperative that providers' discuss the benefit of this vaccine before an AYA MSM sexual debut in lieu of catch up vaccinations as studies have shown that it is effective in in preventing new anal HPV infections (12, 18). Anal dysplasia screening in AYA MSM should be expanded to start at the recommended cervical cancer screening age for women (21 years old); especially since AYA MSM living with HIV may not acknowledge or be aware of their HIV status.

## Author Contributions

JA drafted the initial manuscript and approved the final manuscript as submitted. CG, EW, and SF critically reviewed and revised the initial manuscript and approved the final manuscript as submitted.

## Funding

This article was supported in part by the Health Resources and Services Administration (HRSA) of the U.S. Department of Health and Human Services (HHS) as part of a MCHB T71MC00009 LEAH training grant.

## Author Disclaimer

The contents are those of the authors and do not necessarily represent the official views of nor an endorsement by HRSA, HHS, or the U.S. Government (see HRSA.gov).

## Conflict of Interest

The authors declare that the research was conducted in the absence of any commercial or financial relationships that could be construed as a potential conflict of interest.

## Publisher's Note

All claims expressed in this article are solely those of the authors and do not necessarily represent those of their affiliated organizations, or those of the publisher, the editors and the reviewers. Any product that may be evaluated in this article, or claim that may be made by its manufacturer, is not guaranteed or endorsed by the publisher.
